# Transvaginal Management of Vaginal Cuff Dehiscence with Bowel Evisceration following Delayed Diagnosis

**DOI:** 10.1155/2017/4985382

**Published:** 2017-12-21

**Authors:** Samantha Bleull, Hunter Smith, Robert Shapiro

**Affiliations:** West Virginia University Department of Obstetrics/Gynecology, Morgantown, WV, USA

## Abstract

One of the most serious complications that can arise from hysterectomy is vaginal cuff dehiscence with subsequent bowel evisceration. Treatment via vaginal approach has been utilized in early cases of vaginal cuff dehiscence where the need for bowel resection is less likely. Our case examines the treatment of vaginal cuff dehiscence through a vaginal approach approximately 36 hours after apparent vaginal dehiscence with subsequent bowel evisceration. In this case, we chose a vaginal approach even in the setting of possible bowel obstruction and a significant leukocytosis. We utilized CT scan findings to help guide our surgical approach. Although the subjective appearance of the bowel protruding through the vaginal cuff was reassuring, this played little role in guiding our decision with regard to surgical approach. Vaginal cuff dehiscence with evisceration can be managed successfully via a vaginal approach even with prolonged exposure of the bowel to vaginal flora. CT scan should be utilized to evaluate bowel integrity when considering a vaginal dehiscence repair. A high index of suspicion is warranted as these cases can present up to many years after hysterectomy.

## 1. Introduction

Hysterectomy is the most commonly performed gynecological procedure in the United States [1]. One of the most serious complications that can arise from hysterectomy is vaginal cuff dehiscence [2]. Vaginal cuff dehiscence is the separation of a vaginal incision that was previously closed at the time of hysterectomy [3]. Its incidence is between  .03 and .28% [3].

Dehiscence of the vaginal cuff after hysterectomy occurs predominantly after coitus in premenopausal women [4]. Transabdominal hysterectomies have the lowest incidence of vaginal cuff dehiscence than other methods of hysterectomies, such as laparoscopic, robotics, or vaginal [5]. Vaginal cuff dehiscence can be associated with eviscerations of the bowel, adnexa, and omentum [5].

In many cases, the management of vaginal dehiscence with evisceration consists of immediate laparotomy to assess the overall integrity of the entire bowel. In some instances, bowel resection is required if ischemia is present. A vaginal approach has been utilized in early cases of vaginal cuff dehiscence where the need for bowel resection is less likely [6]. Our case examines the treatment of vaginal cuff dehiscence through a vaginal approach approximately 36 hours after apparent vaginal dehiscence with subsequent bowel evisceration.

## 2. Case Presentation

Patient is a 39-year-old woman that presented with abdominal and vaginal pain after intercourse the previous night. The patient was having diarrhea and abdominal pain for two days before presenting to the emergency department. She was also having difficulty emptying her bladder. 74 days after total abdominal hysterectomy and bilateral salpingectomy, the patient was given permission 5 weeks from her surgery to resume intercourse.

Patient had 4 previous painful sexual encounters since surgery. Past medical history was significant for mitral valve prolapse. Patient had a history of 3 previous cesarean sections, dilation and curettage, and endometrial ablation.

Patient initially presented to a small, community hospital for evaluation. On gynecologic exam at that facility, she was thought to have a moderate cystocele. Abdominal X-ray and computerized tomography (CT) with and without contrast showed possible small bowel obstruction. She was referred to our tertiary care center for possible surgical intervention to relieve the obstruction.

General surgery was consulted upon arrival to our institution. Recommendations were to initially manage conservatively with nasogastric suctioning and bowel rest.

Due to history of prior abdominal hysterectomy, the gynecology service was consulted. Speculum exam showed yellow tinged fluid coming from vaginal cuff and bowel was visualized through vaginal cuff with patient valsalva. Patient had a white blood cell count of 20.1 × 10^3^ uL but was afebrile at admission.

Patient was started on intravenous (IV) clindamycin and gentamycin. She was taken immediately to the operating room. A weighed speculum was placed in the vaginal introitus. Pink and moist small bowel was noted in the vaginal canal. There was no evidence of necrosis or inflammatory changes. The bowel was reduced and the vaginal cuff was trimmed and closed with interrupted figure-of-eight sutures using 0 PDS. Cystoscopy was performed showing normal ureteral orifices, normal bladder architecture, and no evidence of intravesicular damage. A malecot drain was placed through the vagina and into the peritoneal cavity. On postoperative day one, the vaginal drain spontaneously came out. The patient was kept on IV antibiotics for a total of 48 hours and remained afebrile. She was discharged from the hospital on postoperative day 3.

Patient presented at 4-week follow-up doing well with the exception of occasional bladder spasms. At four-month follow-up, the vaginal cuff was well healed with normal caliber and depth. She was given permission to resume sexual relations. At 7-month follow-up, the patient was having occasional left sided pelvic pain exacerbated by intercourse. The vagina had good caliber and length. She began working with a pelvic floor physical therapist which has helped significantly with her pain during intercourse. She continues to do well.

## 3. Discussion

Vaginal cuff dehiscence with or without bowel evisceration has been reported as early as 3 days [7] and as late as 30 years postoperatively [8]. In a particular study which combined and analyzed case reports, the mean time to cuff dehiscence was 13 weeks for patients having an abdominal hysterectomy [9]. This is very much in line with our observations regarding this case.

The precipitating event in our case was vaginal intercourse. According to available literature, this is most common [10]. However, spontaneous vaginal cuff dehiscence can also occur in large proportion of patients [10].

To our knowledge, this is the first case report that describes a vaginal approach for treatment of dehiscence with prolonged bowel evisceration and possible obstruction. Traditionally, repair of vaginal cuff dehiscence with evisceration is performed via laparotomy to ensure bowel integrity. Growing evidence, albeit mostly from anecdotal case reports, continues to emerge suggesting that a minimally invasive approach can be utilized depending on the severity of the patient's symptoms.

Typically, the vaginal route is reserved either for cases that do not involve bowel evisceration or when evisceration is diagnosed immediately before bowel ischemia or sepsis occurs [11]. In this case, we chose a vaginal approach even in the setting of possible bowel obstruction and a significant leukocytosis. Advantages to this approach include shorter hospitalization and postoperative recovery times compared to an abdominal incision [12]. The obvious disadvantage is the inability to fully visualize the bowel and other pelvic viscera that may require surgical intervention.

**Figure 1:**
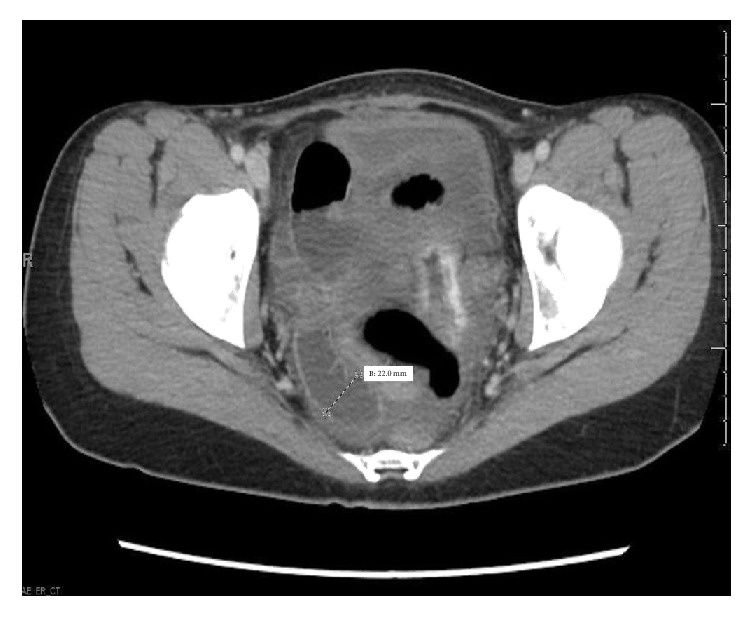
Axial CT image of the small bowel extending into the vagina. Normal thickness is less than 30 mm.

**Figure 2:**
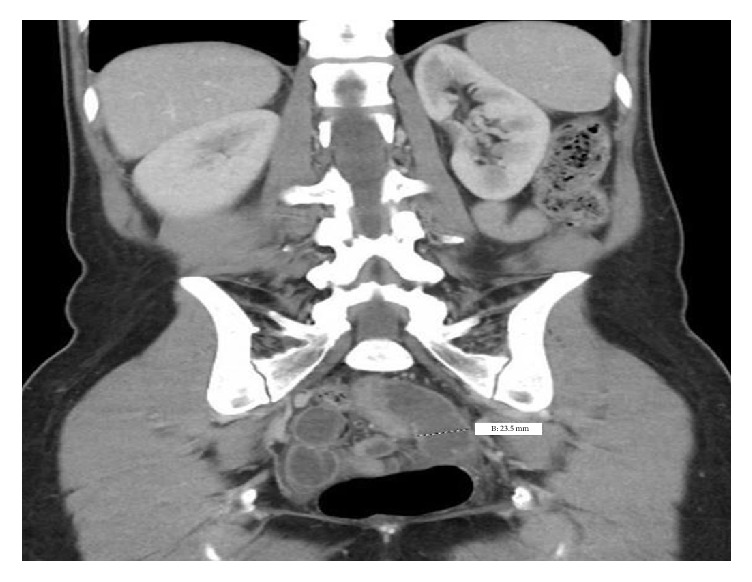
Coronal CT image of the small bowel extending into the vaginal. Normal thickness is less than 30 mm.

We utilized CT scan findings to help guide our surgical approach. The CT findings did not indicate bowel wall thickening, engorgement of mesenteric vessels, or mesentery edema, any of which could imply bowel ischemia [13]. (Please see Figures 1 and 2.) Because of this, we felt confident the integrity of the bowel was intact and a vaginal approach could be utilized. Also, with the exception of leukocytosis, the patient did not exhibit any other signs of evolving sepsis such as fever or tachycardia. Diagnostic cystoscopy was performed to make sure no bladder injury occurred during the repair.

Currently within the literature, no clear consensus exists on how best to surgically approach vaginal cuff dehiscence. The approach often depends on many variables such as bowel involvement, patient stability, and surgeon experience. The unique aspect of our case was the vaginal cuff dehiscence initially presented as a bowel obstruction that led to a delay in diagnosis. This delay could have compromised the integrity of the bowel leading many surgeons to approach this case abdominally.

Although the subjective appearance of the bowel protruding through the vaginal cuff was reassuring, this played little role in guiding our decision with regard to surgical approach. Clinical features of bowel viability such as color and peristalsis have been shown to have little correlation with bowel survival [14].

## 4. Conclusions

1. Vaginal cuff dehiscence with evisceration can be managed successfully via a vaginal approach even with prolonged exposure of the bowel to vaginal flora.
2. CT scan should be utilized to evaluate bowel integrity when considering a vaginal dehiscence repair.
3. A high index of suspicion is warranted as these cases can present up to many years after hysterectomy.
